# Identification of microduplications at Xp21.2 and Xq13.1 in neurodevelopmental disorders

**DOI:** 10.1002/mgg3.1703

**Published:** 2021-05-12

**Authors:** Hannaleena Kokkonen, Auli Siren, Tuomo Määttä, Magda Kamila Kadlubowska, Anushree Acharya, Liz M. Nouel‐Saied, Suzanne M. Leal, Irma Järvelä, Isabelle Schrauwen

**Affiliations:** ^1^ Northern Finland Laboratory Centre NordLab and Medical Research Centre Oulu University Hospital and University of Oulu Oulu Finland; ^2^ Kanta‐Häme Central Hospital Hämeenlinna Finland; ^3^ Disability Services Joint Authority for Kainuu Kajaani Finland; ^4^ Center for Statistical Genetics Sergievsky Center Department of Neurology Columbia University Medical Center New York NY USA; ^5^ Taub Institute for Alzheimer's Disease and the Aging Brain Columbia University Medical Center New York NY USA; ^6^ Department of Medical Genetics University of Helsinki Helsinki Finland

**Keywords:** exome sequencing, intellectual disability, microduplication, neurodevelopmental disorders, X‐chromosome

## Abstract

**Background:**

Microduplications are a rare cause of disease in X‐linked neurodevelopmental disorders but likely have been under reported due challenges in detection and interpretation.

**Methods:**

We performed exome sequencing and subsequent microarray analysis in two families with a neurodevelopmental disorder.

**Results:**

Here, we report on two families each with unique inherited microduplications at Xp21.2 and Xq13.1, respectively. In the first family, a 562.8‐kb duplication at Xq13.1 covering *DLG3*, *TEX11*, *SLC7A3*, *GDPD*2, and part *KIF4A* was identified in a boy whose phenotype was characterized by delayed speech development, mild intellectual disability (ID), mild dysmorphic facial features, a heart defect, and neuropsychiatric symptoms. By interrogating all reported Xq13.1 duplications in individuals affected with a neurodevelopmental disorder, we provide evidence that this genomic region and particularly *DLG3* might be sensitive to an increased dosage. In the second family with four affected males, we found a noncontinuous 223‐ and 204‐kb duplication at Xp21.2, of which the first duplication covers exon 6 of *IL1RAPL1*. The phenotype of the male patients was characterized by delayed speech development, mild to moderate ID, strabismus, and neurobehavioral symptoms. The carrier daughter and her mother had learning difficulties. *IL1RAPL1* shows nonrecurrent causal structural variation and is located at a common fragile site (FRAXC), prone to re‐arrangement.

**Conclusion:**

In conclusion, we show that comprehensive clinical and genetic examination of microduplications on the X‐chromosome can be helpful in undiagnosed cases of neurodevelopmental disease.

## Graphical abstract

Here, we report on two families each with unique inherited microduplications at Xp21.2 and Xq13.1, respectively. In the first family, a 562.8 kb duplication at Xq13.1 covering DLG3 and part of KIF4A. In the second family with 4 affected males, we found a non‐continuous 223 kb and 204 kb duplication at Xp21.2, of which the first covers exon 6 of IL1RAPL1.

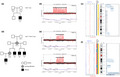

## 1 INTRODUCTION

Neurodevelopmental disorders (NDDs) are genetically heterogeneous conditions. Based on the current, estimates about 30%–40% of NDDs are caused by variants identified by exome sequencing (ES) and 15%–20% by microdeletions or duplications detected by chromosomal microarray (CMA) analyses (Srivastava et al., 2019). X‐linked intellectual disability (ID) is responsible for 10%–12% of male ID cases, and of them, structural variants (SVs) have been found in 5%–15% of cases (Whibley et al., 2010).

Microduplications on the X chromosome are challenging to detect and interpret. However, a careful follow up and investigation of these microduplications may lead to a molecular diagnosis. When screening Finnish families with cases of unexplained NDDs using ES, we identified two families with possible X‐chromosomal duplications in areas with known NDD genes. The variants were further characterized and confirmed using chromosome microarray analysis (CMA). Here we report the detailed clinical phenotypes and molecular genetic analyses of the identified families.

## 2 MATERIALS AND METHODS

### 2.1 Ethical compliance

Written informed consent was obtained from healthy adult subjects and the parents/legal guardians of minor subjects and ID patients. The study was approved by the ethics committees of the Hospital District of Helsinki and Uusimaa and the Institutional review board of Columbia University, New York (IRB‐AAAS3433).

### 2.2 Exome sequencing

**FIGURE 1:**
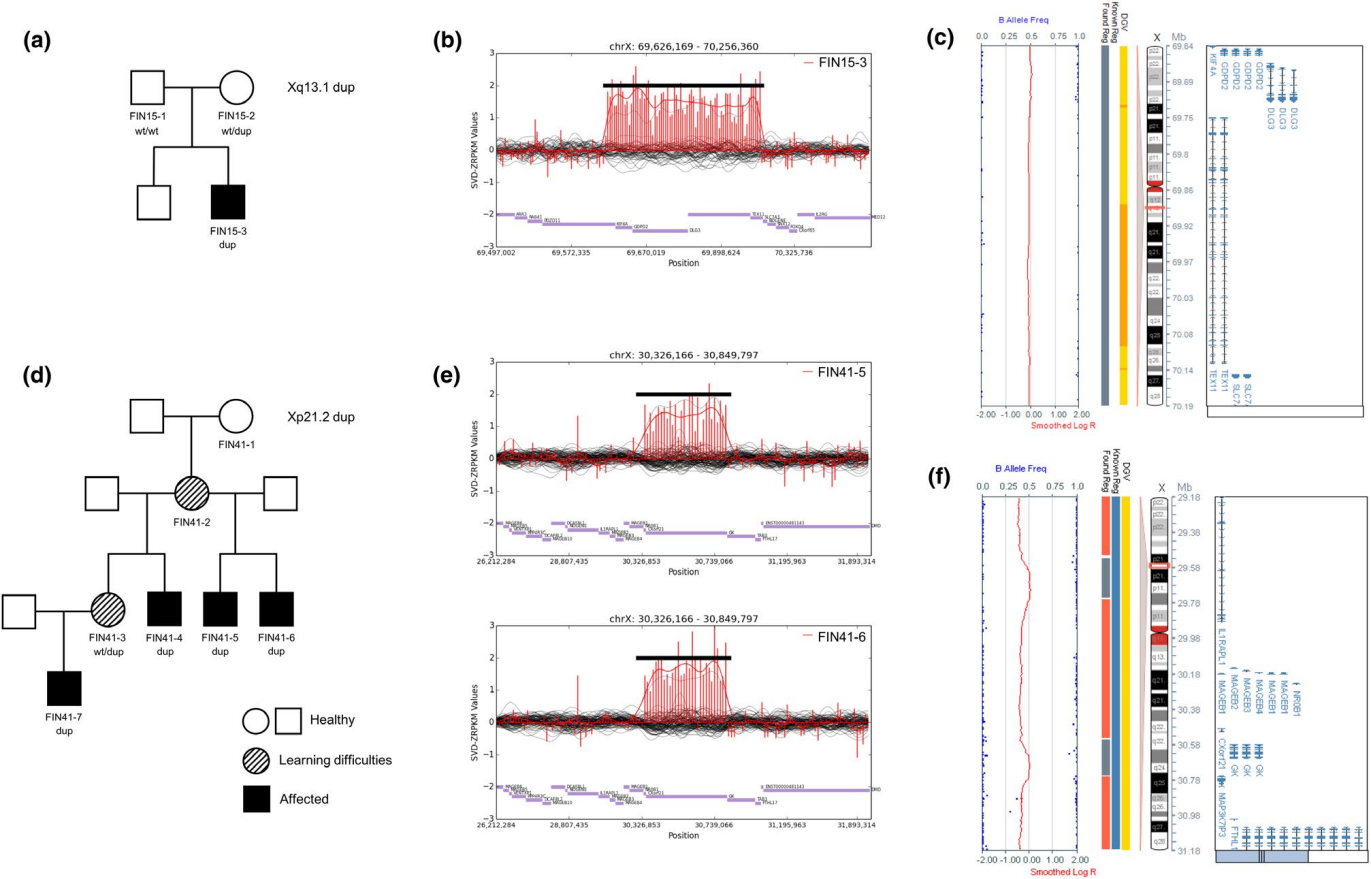
Pedigrees and duplications identified in this study. (a) Pedigree of FIN15 in which a microduplication at Xq13.1 found via ES (631 kb) (b) and validated via CMA (562.8 kb; (c and d) Pedigree of FIN41, in which a 524‐kb microduplication at Xp21.2 identified via ES (e) and validated further with CMA showing two noncontinuous microduplications, a 223‐kb duplication at Xp21.2 and a 204‐kb duplication at Xp21.2 (f)

Genomic DNA was extracted from peripheral blood using the NucleoSpin blood XL kit (Macherey Nagel, Germany), according to the manufacturer's instructions. DNA samples from trio FIN15 (FIN15‐1; FIN15‐2 and FIN15‐3) and from family FIN41 (FIN41‐5 and FIN41‐6) underwent exome sequencing (Figure 1). Target enrichment was done using the SureSelect Human All Exon V6 kit, and paired‐end sequencing was performed on a HiSeq2500/4000 instrument (Illumina Inc, San Diego, CA, USA). Bioinformatic details can be found in the supporting information. In short, data were aligned to the human genome, single nucleotide variant (SNV), Insertion/Deletions (InDels), and Copy number variants (CNV) were called and annotated. Rare SNV, InDel, and CNV variants that fit the appropriate inheritance models based on the pedigree and were predicted to have a functional effect on gene function were retained.

### 2.3 Microarray analysis

We validated candidate CNVs detected via exome sequencing and tested segregation via a CMA in family members. Microarray analysis was performed using a 50mer‐oligochip (HumanCytoSNP‐12v2.1, Illumina Inc.) that allowed an effective resolution as small as 30 kb in cytogenetically relevant regions and 200 kb in other areas of the genome. Copy‐number changes and regions of SNV‐homozygosity were analyzed with GenomeStudio v.2011.1 and KaryoStudio 1.3 programs (Illumina Inc.) using reference genome GRCh37/hg19. Identified CNVs were compared with known CNVs listed in the DGV, dbVar, UCSC genome browser, DECIPHER, and OMIM databases and further interrogated using peer‐reviewed literature searches in the PubMed database.

### 2.4 Review of reported copy number variants identified at Xq13.1 and Xp21.2 in individuals with a NDD

To identify reported duplications on Xq13.1 associated with a NDD, we interrogated Pubmed, the Columbia University catalog and DECIPHER (Firth et al., 2009). The criteria used were the following: (1) duplications with overlap with the duplication from the current study, (2) exact genomic coordinates had been determined, (3) length under 10 Mb, (4) phenotypic overlap between cases, (5) males only were included, and (6) individuals with other genomic variants likely implicated in their disease were excluded.

For Xp21.2, a literature search using Pubmed and the Columbia University catalog was performed to identify intragenic variants *IL1RAPL1* (**#** 300143; MRX21) previously associated with neurodevelopmental disease. (1) Both intragenic microduplications and deletions in *IL1RAPL1* were identified (<1.3 Mb, the size of *IL1RAPL1*). (2) Only variants with detailed coordinates were retained. (3) Individuals with a phenotypic similarity with our patient were retained, including ID and developmental delay, typical for the disorder associated with *IL1RAPL1* (Mental retardation, X‐linked 21/34). (4) Individuals with other genomic variants likely implicated in their disease were excluded.

## 3 RESULTS

Clinical features and molecular genetic findings are shown in Table 1 and in Figure 1. Detailed clinical descriptions of the families are provided in the supporting information.

**TABLE 1:** Clinical details and molecular genetic findings of the study subjects

	Age at diagnosis	Sex	Delayed speech and motor development	Learning disability	Neuropsychiatric/ neurobehavioral symptoms	ID	Other phenotypic features	X‐chromosomal duplication	Other CMA findings
FIN15‐3	28 years	M	Yes	Yes	Distractability, anxiety, psychosis	Mild	syndromic facial features, cardiac defects	Xq13.1 dup	No
FIN41‐2	46 years	F	No	Mild	ND	ND	No	ND	No
FIN41‐3	26 years	F	No	Mild	No	ND	No	Xp21.2 dups	13p13.3dup
FIN41‐4	22 years	M	Yes	Severe	Distractability, impulsive behaviour	Moderate	No	Xp21.2 dups	7p15.5dup; 13p13.3dup
FIN41‐5	15 years	M	Yes	Severe	distractability, impulsive behaviour	Moderate	No	Xp21.2 dups	13p13.3dup
FIN41‐6	12 years	M	Yes	Severe	Distractability, impulsive behaviour	Moderate	No	Xp21.2 dups	13p13.3dup^a^
FIN41‐7	8 years	M	Yes	Severe	Distractability, impulsive behaviour	Mild	No	Xp21.2 dups	13p13.3dup

Abbreviations: disability; ID, intellectual; ND, not data.

^a^ Noncontinuous rearranged duplication compared to other family members. For more details see supporting information.

### 3.1 Family FIN15 shows a microduplication at Xq13.1 and *DLG3* may be sensitive to an increased dosage

In family FIN15, a 631‐kb duplication at Xq13.1 [chrX:69625698–70256360, hg19; NC_000023.10:g.(?_69625698)_(70256360_?)dup] was found via ES in the affected son (Figure 1a–c). The patient showed mild ID, dysmorphic facial features, transposition of the great arteries, coarctation of the aorta, and psychotic behavior. His mother was a carrier of the duplication. The finding was confirmed using microarray analysis where a 562.8‐kb duplication at Xq13.1 [chrX:69637865–70220983, hg19; NC_000023.10:g.(?_69637865)_(70220983_?)dup] was identified covering *DLG3* (**#** 300850), *TEX11* (**#** 309120*)*, *SLC7A3* (*#* 300443*)*, *GDPD2* (**#** 300940) genes, and at least exons 30–31 of the *KIF4A* gene (**#** 300923) (Figure 1c). The mother has long‐QT syndrome, caused by a known pathogenic variant in *KCNQ1* (NM_181798.1: c.1385G>A:p.G462D) (**#** 192500); not present in her son) but is otherwise healthy. The father did not have the duplication. In addition, the index patient has two compound heterozygous variants in *CNTN1* (**#** 612540) and *KIF26A* (**#** 613231), classified as variants of unknown significance (VUS) (Table S1).

**FIGURE 2:**
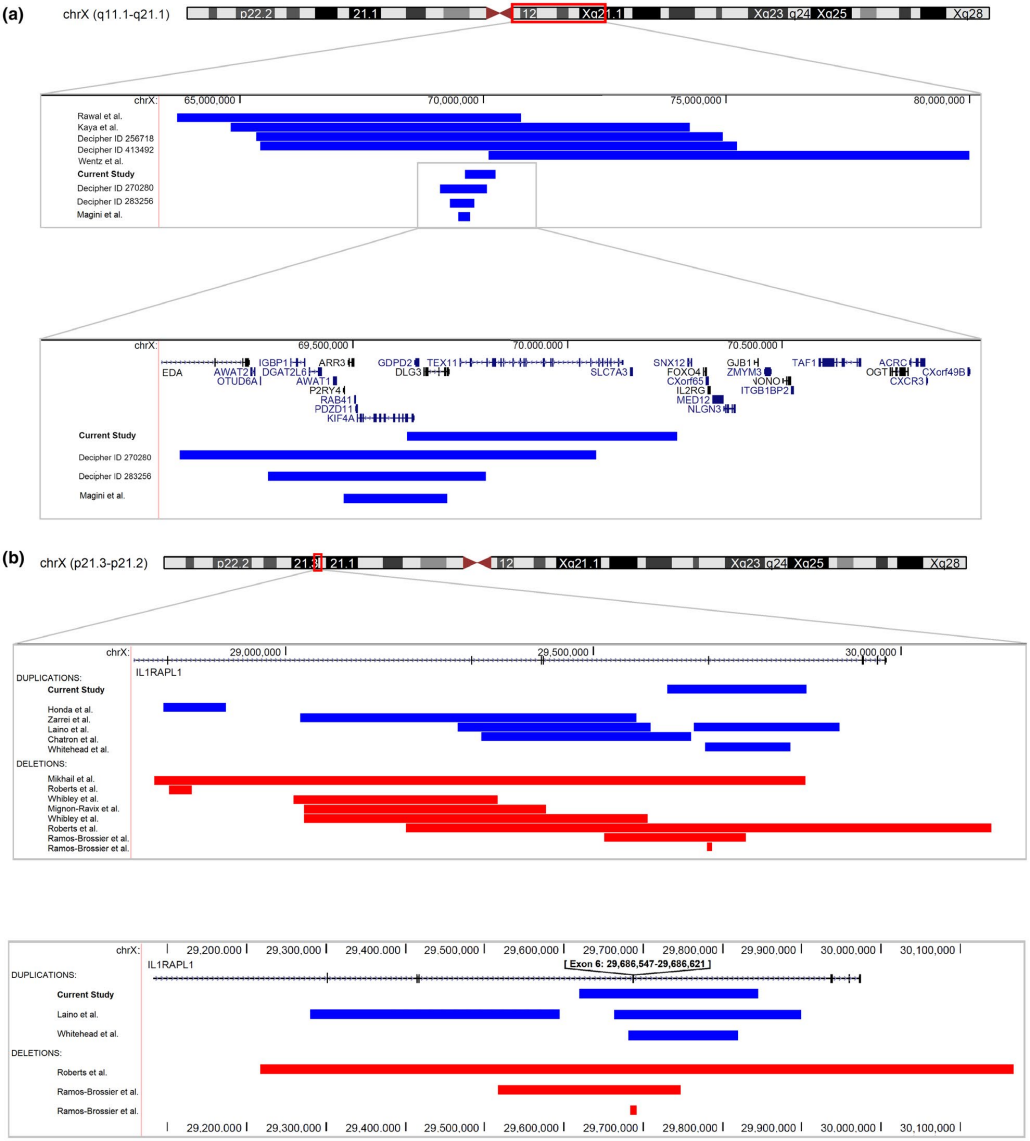
Copy number variants (CNVs) identified at Xq13.1 (a) and Xp21.2 (b) in the literature in males affected with neurodevelopmental disorders with overlapping features. (a) Microduplications (blue) identified at Xq13.1 in affected individuals with overlapping features. This and additional microduplications in this region in DECIPHER (Firth et al., 2009) suggest an increased dosage sensitivity for *DLG3*. (b) Intragenic microduplications (blue) and deletions (red) in *IL1RAPL1* (Xp21.2) identified show a variable profile of nonrecurrent CNVs in *IL1RAPL1*. The bottom panel shows exon 6 microdeletions and duplications only. Methods and more details are available in the supporting information

We next reviewed all cases with a NDD with overlapping features and duplications at Xq13.1, as shown in Table S2 and Figure 2. Via a literature search, we identified three duplications in this area in eight male patients with phenotypic overlap including ID, abnormal behavior and dysmorphic features (Kaya et al., 2012; Bhattacharya et al., 2019; Wentz et al., 2014), and a microduplication in two brothers with a NDD with seizures only (Magini et al., 2019). Seven additional overlapping duplications in patients with phenotypic overlap are currently present in DECIPHER, of which four are shown in Figure 2 for which permission was obtained (Firth et al., 2009). Figure 2 shows that this genomic area might be sensitive to increased dosage. This might particularly be true for *DLG3*, which is present in a minimal overlapping region. Single nucleotide and InDel variants in *DLG3* are known to cause a NDD with variable degrees of ID, dysmorphic features, language delay, and epilepsy in some cases (Philips et al., 2014; Tarpey et al., 2004). Magini et al. also suggested that the microduplication they identified in two affected siblings with epilepsy may be due to increased dosage sensitivity of *DLG3* and/or *KIF4A* (Magini et al., 2019). Our data provide more evidence to show that *DLG3* is sensitive to an increased dosage and associated with a similar phenotype as SNV/InDel variants.

### 3.2 Family FIN41 shows a non‐continuous microduplication at Xp21.2 covering exon 6 of *IL1RAPL1*

A 524‐kb microduplication at Xp21.2 [chrX:30326166–30849797, hg19; NC_000023.10:g.(?_ 30326166)_(30849797_?)dup] was identified in both FIN41‐5 and FIN41‐6 via ES (Figure 1d–f). In CMA analysis, the region showed two closely neighboring microduplications; a 223‐kb duplication at Xp21.2 (chrX:29619835–29843303, hg19) and a 204‐kb duplication at Xp21.2 (chrX:30646799–30848980, hg19) were found in the carrier female (FIN41‐3) and in four affected males (FIN41‐4 ‐FIN41‐7) (Table 1 and Figure 1f) (NC_000023.10:g.[(?_29619835)_(29843303_?);(?_30646799)_(30848980_?)dup]). The phenotype of the affected males was characterized by delayed language development, mild to moderate ID and neurobehavioral changes. The carrier female (FIN41‐3) and her mother (FIN41‐2) had learning difficulties at school. Based on the molecular genetic findings, FIN41‐2 is likely a carrier of the duplication, although her sample was not available.

The Xp21.2 duplication includes exon 6 (NM_014271.3) of *IL1RAPL1* (# 300206), interleukin‐1 receptor accessory protein like 1 gene, which could possibly affect the function of its extracellular domain. The in‐frame duplication of exon 6 is predicted to lead to an insertion of 25 amino acids in the extracellular domain of *IL1RAPL1*, between immunoglobulin domain (Ig) 2 and 3 p.(Ala235_Leu259dup). *IL1RAPL1* is mostly expressed in the brain where it regulates synapse formation, and it has activity on synaptogenesis and dendrite morphology (Montani et al., 2019; Ramos‐Brossier et al., 2015).

The proximal duplication at Xp21.2 (chrX:30643031–30847448, hg19) covers the glycerol kinase (*GK*) gene (**#** 300474) and the part of *TAB3*‐gene (**#** 300480). SNVs, InDels, and large deletions in the GK gene underlie XLR Glycerol kinase deficiency (GKD) (OMIM #307030) known to cause a metabolic disorder. GKD results in hyperglycerolemia, a condition characterized by the accumulation of glycerol in the blood and urine. Isolated glycerol kinase deficiency is believed to be a benign condition (FM Vaz, personal communication).

All four affected males have a similar nonsyndromic phenotype with mild to moderate ID, strabismus and hyperactivity (Table 1). The first symptom was delayed speech development identified at 2–3 years of age. The two carrier females, of them FIN41‐3 molecularly tested, only showed learning difficulties.

In addition, two other microduplications at 13q13.3 and 7p15.5 were detected in the FIN41 family (Table 1; supporting information). ES also showed that FIN41‐5 and FIN41‐6 had possible compound heterozygous variants in *NPHP4* (**#** 606966), which were classified as VUS (Table S1).

Via a literature search, we retrieved 13 intragenic CNVs in *IL1RAPL1* from 10 studies (Chatron et al., 2017; Honda et al., 2010; Laino et al., 2016; Mignon‐Ravix et al., 2014; Mikhail et al., 2011; Ramos‐Brossier et al., 2015; Roberts et al., 2014; Whibley et al., 2010; Whitehead et al., 2016; Zarrei et al., 2019), including 6 variants, 3 deletions, and 3 duplications, covering exon 6 (Laino et al., 2016; Ramos‐Brossier et al., 2015; Roberts et al., 2014; Whitehead et al., 2016). This showed that no recurrent variants were found intragenically. An overview of the variants can be found in Figure 2 and an overview of phenotypic features in Table S3.

## 4 DISCUSSION

In this study, we report two families with different inherited X‐linked duplications. In the first family, a duplication at chrXq13.1 was first detected using ES, which was confirmed via CMA to be 563 kb. In the duplicated region Xq13.1, two genes, *DLG3* and *KIF4A* (partial), are known to cause ID. We and others have reported SNV and InDel variants in *DLG3* (Philips et al., 2014; Tarpey et al., 2004) in cases characterized by mild to severe ID, mild dysmorphic features, language delay, and epilepsy in some patients. *KIF4A* is involved in cell division and has been reported to underlie mild to moderate nonsyndromic ID, language delay, and epilepsy. *TEX11*, *SLC7A3*, and *GDPD2* have not been associated with brain function (www.omim.org). Via a detailed analysis of reported cases with duplications at Xq13.1, we provide evidence that *DLG3* may have an increased dosage sensitivity in this region (Figure 2). We show that microduplications at Xq13.1 are also associated with a similar phenotype as the phenotype due to SNV/InDel variants in *DLG3* (Table S2).

In the second family, a Xp21.2 duplication was identified in four affected males and one female. Single nucleotide, InDel, and copy number variants in *IL1RAPL1* have been associated with a variable phenotype ranging from nonsyndromic ID to autism spectrum disorder (ASD) (Ramos‐Brossier et al., 2015). The identified Xp21.2 duplication contains exon 6 of the *IL1RAPL1* gene, is predicted to lead to p.(Ala235_Leu259dup), and is expected to have a similar effect as other intragenic microCNVs covering exon 6 (Figure 2; Table S3) (Philips et al., 2014; Tarpey et al., 2004). Affected males with exon 6 duplications and deletions indeed show similar features including language and motor development delay and ID (Table S3).

Female carriers of the exon 6 duplication in our family showed a mild phenotype of learning disability. Similarly, three female carriers of an exon 6 deletion in *IL1RAPL1*, predicted to lead to p.(Ala235_Leu259del), also had learning difficulties or ID only (Table S3) (Ramos‐Brossier et al., 2015). This is not surprising as a female carrier phenotype is present in the majority of X‐linked ID disorders, although this is often a milder phenotype (Ziats et al., 2020). One of the females with an exon 6 deletion in *IL1RAPL1* showed random X‐inactivation based on studies in her fibroblast cells (Ramos‐Brossier et al., 2015).

Intragenic deletions of *IL1RAPL1* are a common disease mechanism (Whibley et al., 2010); however, intragenic disease‐associated duplications in this gene are less common (Laino et al., 2016b). The mechanisms of *IL1RAPL1* rearrangement are likely related to its presence in the common fragile site *FRAXC*, and the implicated mechanisms of SV creation may favor deletions (Whibley et al., 2010). Common fragile sites are common regions of profound genomic instability. *FRAXC* is a common fragile site containing both *DMD* and *IL1RAPL1*; both are genes in which SVs are often involved in Mendelian disease. Instability‐induced alterations will primarily occur within intronic regions, and *IL1RAPL1* covers a large genomic region (1.37 Mbs) with >99% of its sequence intronic. *IL1RAPL1*'s large genomic size in an area of instability makes it susceptible to DNA breakage and gene rearrangements. Of interest, we also identified a second duplication adjacent to *IL1RAPL1* at Xp21.2 in our patient. Similar to previous reports of noncontinuous microSVs in this region (Chatron et al., 2017; Laino et al., 2016b), this is also likely due to this area being prone to breakage and subsequent incorrect rearrangement.

As the duplication we identified in *IL1RAPL1* is nonrecurrent (Figure 2) nor is there significant homology between introns (no low copy repeats), nonhomologous end joining (NHEJ) and microhomology‐mediated mechanisms might be the more likely mechanism in our case. Although the exact breakpoints of the duplication in *IL1RAPL1* are unknown, the breakpoint sites do primarily contain long interspersed nuclear elements (*LINE*) elements and some *Alu* repeats. The disproportionate rate of deletions relative to duplications has also been seen at some nonallelic homologous recombination (NAHR) hotspots (Turner et al., 2008), and *Alu*‐*Alu*‐ or *LINE*‐*LINE*‐mediated NAHR may also have occurred. Similar to the Xp21.2 duplication, the Xq13.1 duplication is also nonrecurrent (Figure 2), does not contain any low copy repeats, and the breakpoint areas are flanked by a large number of *Alu* repeats and *LINE* repeats as well, suggesting NEJH or microhomology‐mediated mechanisms.

In conclusion, our study shows that exome sequencing is a useful tool to screen for microCNVs, which can lead to a molecular diagnosis via additional molecular testing and research. By combining careful clinical analysis, literature and further genetic characterization, we report two novel microduplications on chromosome X implicated in X‐linked ID and provide evidence that *DLG3* is sensitive to increased dosage.

## CONFLICT OF INTEREST

The authors declare no conflict of interests.

## AUTHOR CONTRIBUTIONS

I.J and I.S. conceived and planned the project; I.J and I.S. wrote the manuscript with contributions from A.S., HL.K., and S.L.; A.S. and T.M. recruited the cases; A.S., T.M., and I.J. examined the cases; A.S. and I.J. collated and analyzed the clinical data; I.S., A.A., and L.N. performed exome sequencing data analysis; HL.K. performed genomic copy number analysis; M.K. drew the figures and performed a literature research.

## Supporting information

Table S1

Table S2

Table S3

Supplementary Material

## Data Availability

Variants have been deposited into ClinVar (Accession numbers: SCV001451899.1 and SCV001451900.1.
