# Supplementary material for: Identification of microduplications at Xp21.2 and Xq13.1 in neurodevelopmental disorders
Source: Mol Genet Genomic Med. 2021 May 12;9(12):e1703. doi: 10.1002/mgg3.1703 (PMC8683627; doi:10.1002/mgg3.1703)
Supplement: Supplementary file 4 — Supplementary Material [file MGG3-9-e1703-s002.docx]

**Supplementary data**

***Clinical description of the patients and families***

**FIN15-3** is a 28 years old male, II/2 child of the family (Figure 1A). His birth weight was 4460 g. He learnt to walk at 1 y of age and speak at 3 y of age. He was operated due to transposition of the great vessels and coarctation of aortae soon after the birth. At school age clumsiness and learning difficulties were noticed. Facial features include hypotelorism, small nose, small mouth and short neck. His major challenges are short attention span, distractibility, a high level of anxiety and psychotic episodes that limit his daily activities and further education. His brain MRI was normal.

**FIN41-1** is a healthy grandmother who takes care of FIN41-4.

**FIN41-2** has one daughter with borderline intellectual functioning and three sons from two marriages with a similar neurodevelopmental disorder (Figure 1D). Her learning difficulties were noticed at school age. She did not attend any further education and has not had permanent work.

**FIN41-3,** like her mother had learning difficulties that were detected at school age and she obtained special education. She graduated herself as a hairdresser but stayed at home until starting to educate herself as a helper for elderly people. Her intellectual level is borderline normal intellectual functioning.

**FIN41-4** is II/4 child of FIN41-2. He was born after normal pregnancy and delivery, birth weight 3900g, height 51 cm, Apgar 8/9. He was studied due delayed psychomotor development at 1.5 years of age. He learned to walk at the age of 1.5 y and at that point his cognitive skills were around 11 months. Karyotype, *FMR1* genetic testing, EEG and urine metabolic screening (amino acids, mucopolysaccarides, oligosaccarides) were normal. CK was 438 slightly elevated. Visus 0.16/0.5-0.6. He has amblyopia in his right eye, papilla and the macula area are normal with normal VEP and ERG. He has got speech therapy and obtained extended special education. He cannot read. He was restless, and needed a lot of support at school. At age of 9 years he was studied due impulsive behavior. He has moderate ID. His adaptive daily skills were good for the age, but he needed a lot surveillance. He is not able to live independently.

**FIN41-5** is III/4 child of FIN41-2, 15 years old (Figure 1D). His early phases were normal. He learnt to speak his first words at age 1 year and walk independently at 1year 5 months of age. At 3.5 years of age he was studied due delayed psychomotor development. His speech was unclear and clumsiness was detected. Laboratory investigations, CK 164, urine amino acids, oligosaccharides and mucopolysaccharides were normal. EEG, brain MRI, CT, *FMR1* genetic testing and karyotype were normal. Urine creatinine were normal. Since 4 years of age poor impulse control and attention problems were noticed. His daily activity skills are normal. Later he was diagnosed to have ADHD and aggression bursts. He has moderate ID. He is very careful about his own items and he likes to keep his things well organized. To control his ADHD he had several medications. Many of them caused side effects such as methylphenidate loss of appetite, atomoksetine repeated paleness and weakness episodes, risperidon daytime enuresis and elevated blood pressure, aripiprazole somnolency, slow speech, and abnormal tiredness. During guanfacine he had two episodes of hypotonia, and prolonged abnormal deep sleep periods and somnolency. Pharmacogenetic testing of cytochrome enzymes showed no abnormalities. He had special education and lives in specialized facilities for persons with ID.

**FIN41-6** is 12 years old, the IV/4 child of FIN41-2. His early phases were normal. He was referred to pediatric neurologists at 2 years of age due to delayed psychomotor development. He learned to walk at the age 1 year 10 months. He has esotropia. Basic laboratory investigations were normal. At 9 years of age ADHD was diagnosed and methylphenidate started with good effect. His cognition was at the 6-8 y age level when he was 12.5 years referring to moderate ID. He has learned to read slowly. His behavior is often challenging due to impulsivity.

**FIN41-7** is 8 years old son of FIN41-3. He was born at 41+2 weeks of pregnancy. He had mild asphyxia at delivery. His birth weight was 4270, height 54 cm, Apgar 5/8. Ultrasound of brain and abdomen were normal. He was evaluated due delayed development at pediatric neurologist. He learned to walk at the age of 2 years and the first words after age of 2 years. Due to language delay speech therapy was started. Brain MRI, EEG and FMR1 genetic test were normal. He had attention difficulties at day care, and he benefitted of picture communication to support understanding. He has mild strabismus. He has obtained special education. He has mild ID evaluated at the age of 6 years.

**Additional CNVs detected in FIN41**

A 13q13.3 duplication (chr13:36581357-37479138, hg19) was found in several members of the FIN41 family (Table 1), in FIN41-6 divided in two pieces [(13p13.3dup (chr13:36581357-36802675); 13p13.3dup (chr13:37291294-37479138)]. The region contains eight protein coding genes, of them three disease causing (SPG20; Troyer syndrome 275900), *RFXAP* (Bare lymphocyte syndrome type II, complementation group D, 209920) and *SMAD9* (primary pulmonal hypertension, 615342). Proximal breakpoint (chr13:36590118=cnv2/ chr13:36597840=cnv3) is located inside the *DCLK1* gene (chr13:36342789-36705514, hg19). *DCLK1* is a haploinsufficient gene (HI% = 6.47), that is involved in synaptic plasticity and it’s variants are associated with cognition, verbal memory and neuropsychiatric diseases.^1^ The 13q13.3-duplication is not known previously and a partial duplication of *DCLK1* is of unknown significance.

At 7p15.5 a 330 kb duplication containing part of the *CHN2*-gene (chr7:29209361-29536707, hg19) was found in FIN41-4, reported to be associated with lymphomas and schizophrenia. Four similar duplications have been mentioned in Decipher database,^2^ of them three classified as VUS (patient 275779, 261059 and 300210) and one likely benign (patient 288047). As the change has been found four times in control population (1/2026, 3/17000, DGV2), its significance remains unknown.

***Supplementary methods***

**Exome sequencing**

Target enrichment was done using the SureSelect Human All Exon V6 kit and 100 bp paired-end sequencing was performed on a HiSeq2500/4000 instrument (Illumina Inc, San Diego, CA, USA). The average on-target sequencing depth was 96x. Data was aligned to the human genome (GRCh37/Hg19) using Burrows-Wheeler Aligner-MEM (BWAv0.7.15)^3^ and duplicate marking, insertion/deletion (InDel)-realignment, base quality score recalibration and single nucleotide variant (SNV) and InDel calling were performed with Picard-tools (v2.5.0) and the Genome Analysis Toolkit (GATK) (v3.7).^4^ Sample sex and family relations were confirmed for each pedigree using plink (v1.90)^5^ and variants were annotated and filtered using ANNOVAR^6^ and custom scripts. Exonic and splice region variants were retained and several inheritance models were considered depending on the pedigree. A population specific minor allele frequency (MAF) cut-off of <0.005 was used (<0.0005 for Autosomal Dominant) based on the Genome Aggregation Database (gnomAD).^7^ Bioinformatic prediction scores from dbnsfp35a and dbscSNV1.1 annotated with ANNOVAR were used to evaluate missense and splice site variants respectively.^6^ Copy number variants (CNV) were assessed using Copy Number Inference from Exome Reads (CoNIFERv.0.2.2).^8^ The database of genomic variants (DGV) and gnomAD were used to identify rare variants with similar MAF cut-offs as mentioned above.^7^ Variants were classified according to the American College of Medical Genetics and Genomics (ACMG) guidelines.^9,10^

***Web Resources***

The URLs for websites referred to herein are as follows: Database of Genomic Variants (http://projects.tcag.ca/variation/) NCBI’s Database of genomic structural variation (dbVar) (http://www.ncbi.nlm.nih.gov/dbvar/) DECIPHER (http://decipher.sanger.ac.uk), ECARUCA (http://umcecaruca01.extern.umcn.nl:8080/ecaruca/ecaruca.jsp) ClinGen: (https://www.clinicalgenome.org/resources-tools/) ClinVar: (https://www.ncbi.nlm.nih.gov/clinvar/) ExAC Browser: (http://exac.broadinstitute.org/)

**References**

1. Håvik B, Degenhardt FA, Johansson S, et al. DCLK1 Variants Are Associated across Schizophrenia and Attention Deficit/Hyperactivity Disorder. *PLoS One*. 2012;7(4). doi:10.1371/journal.pone.0035424

2. Firth HV, Richards SM, Bevan AP, et al. DECIPHER: Database of Chromosomal Imbalance and Phenotype in Humans Using Ensembl Resources. *Am J Hum Genet*. 2009;84(4):524-533. doi:10.1016/j.ajhg.2009.03.010

3. Li H, Durbin R. Fast and accurate short read alignment with Burrows-Wheeler transform. *Bioinformatics*. 2009;25(14):1754-1760. doi:10.1093/bioinformatics/btp324

4. McKenna A, Hanna M, Banks E, et al. The Genome Analysis Toolkit: A MapReduce framework for analyzing next-generation DNA sequencing data. *Genome Res*. 2010;20(9):1297-1303. doi:10.1101/gr.107524.110

5. Chang CC, Chow CC, Tellier LC, Vattikuti S, Purcell SM, Lee JJ. Second-generation PLINK: rising to the challenge of larger and richer datasets. *Gigascience*. 2015;4(1). doi:10.1186/s13742-015-0047-8

6. Yang H, Wang K. Genomic variant annotation and prioritization with ANNOVAR and wANNOVAR. *Nat Protoc*. 2015;10(10):1556-1566. doi:10.1038/nprot.2015.105

7. Lek M, Karczewski KJ, Minikel EV, et al. Analysis of protein-coding genetic variation in 60,706 humans. *Nature*. 2016;536(7616):285-291. doi:10.1038/nature19057

8. Krumm N, Sudmant PH, Ko A, et al. Copy number variation detection and genotyping from exome sequence data. *Genome Res*. 2012;22(8):1525-1532. doi:10.1101/gr.138115.112

9. Richards S, Aziz N, Bale S, et al. Standards and guidelines for the interpretation of sequence variants: a joint consensus recommendation of the American College of Medical Genetics and Genomics and the Association for Molecular Pathology. *Genet Med*. 2015;17(5):405-424. doi:10.1038/gim.2015.30

10. Riggs ER, Andersen EF, Cherry AM, et al. Technical standards for the interpretation and reporting of constitutional copy-number variants: a joint consensus recommendation of the American College of Medical Genetics and Genomics (ACMG) and the Clinical Genome Resource (ClinGen). *Genet Med*. 2020;22(2):245-257. doi:10.1038/s41436-019-0686-8
